# Healthcare disparities amongst vulnerable populations of Arabs and Jews in Israel

**DOI:** 10.1186/s13584-018-0226-z

**Published:** 2018-05-22

**Authors:** Efrat Shadmi

**Affiliations:** 1 Faculty of Social Welfare and Health Sciences, University of Haifa, Haifa, Israel; 2 Department of Health Policy Planning and the Clalit research Institute, Chief Physician Office, Clalit Health Services, Tel Aviv, Israel

## Abstract

The complex nature of studying health and healthcare disparities in general, and in the context of the Israeli healthcare system in particular, is depicted in two recent IJHPR articles. The first examines Emergency Department (ED) waiting times in a tertiary children’s hospital and the second examines disparities in the health care for people with schizophrenia of an ethnic-national minority. Contrary to other Israeli studies on wide disparities in health and healthcare, these studies show no disparities -  ED waiting times did not differ among Arab and Jewish children and report no differences in performance of Hemoglobin A1C tests or in surgical interventions in patients with cardiovascular disease between Arabs and Jews with schizophrenia. Thus, the studies reflect areas of equitable health care delivery within the Israeli healthcare system.

Future studies should account for the fact that the phenomena of health and healthcare disparities is complex and should utilize rigorous methodologies to take into consideration the various factors that may affect the manifestation of differences amongst population groups. As a result, they may help detect disparities which may otherwise be missed.

Two recent Israel Journal of Health Policy Research articles depict the complex nature of studying health and healthcare disparities in general and in the context of the Israeli healthcare system in particular. Feldman and colleagues [1] studied ethnic differences in Emergency Department (ED) waiting times between Jewish and Arab children in a tertiary children’s hospital in northern Israel. Gal and colleagues [2] compare health care for high cholesterol, diabetes and cardiovascular disease in Jews and Arabs with schizophrenia in a cohort of patients of the largest Israeli health care organization.

The two papers present an impressive platform for the study of ethnic disparities within vulnerable population groups, namely children [1] and patients with schizophrenia [2]. Both utilize large cohorts (> 50,000 individuals) from data that was collected over a span of more than five years. Gal and colleagues [2] report that fewer annual visits to specialists were recorded for service users diagnosed with schizophrenia than among their counterparts, and among Arab-Israelis compared to Jewish-Israelis. No differences were found in performance of Hemoglobin A1C tests or in surgical interventions in patients with cardio vascular disease between Arabs and Jews. Feldman and colleagues [1] show that ED waiting times did not differ among Arab and Jewish children. Moreover, their study showed that ethnic concordance between patients and the ED’s triage nurses was not significantly associated with waiting times.

These findings should be evaluated in light of the multifaceted makeup of the Israeli society and its healthcare system, within a comprehensive approach that accounts for the various types of interrelated social determinants of health [3] and the complexity of health [4]. In Israel, Arabs constitute the largest minority group, which comprises about 20% of the population [5]. Arabs in Israel have been shown to present poorer healthcare utilization patterns and worse health outcomes in a wide variety of clinical domains. Life expectancy is consistently lower among Israeli Arabs than Israeli Jews [6]. Israeli studies have shown that Arabs develop heart failure and diabetes at a much younger age compared with their Jewish counterparts and have a higher prevalence of diabetes and obesity [7, 8]. Arabs in Israel generally have lower socioeconomic status than Jews, and more than 50% of the Arab population lives under the national poverty line [9]. Moreover, health literacy, a well-documented risk factor of poor health outcomes, has been shown to be disproportionally present in Arabs versus Jews, partly attributable to language barriers and to lower education levels [10]. Recent research has shown that while Arabs in Israel have poorer self-rated-health than Jews, this relationship is reversed when adjusting for socio-economic status and living environment [11], exemplifying the complex nature of the manifestation of health disparities.

Despite universal coverage and a national health insurance law in Israel since 1995, a wide-array of gaps in health care service use have been extensively documented, including, as also exemplified by Gal and colleagues [2], lower rates of specialist visits among Arabs than among Jews [12] and lower knowledge of attributes of the healthcare system, such as knowledge on supplementary health insurance [13]. For example, Reges and colleagues [14] show that ethnicity (Arab minority) is by far the most significant barrier to participation of patients with acute coronary syndrome in cardiac rehabilitation programs in Israel. Primary care use, on the contrary, has been shown to be higher among Arabs than Jews [12], which may reflect a pro-poor trend observed also in other countries [15].

The null findings regarding disparities in most of the indicators reported by Gal and colleagues [2] may reflect the indirect effect of the equity promoting mechanisms in the Israeli healthcare system, such as accessible and professional primary care services, which play a pivotal role in promoting equity in chronic disease management, and targeted programs to reduce disparities in primary care [16, 17]. A few additional Israeli studies have reported on lack of disparities or even on pro-minority differences in care between Arabs and Jews. For example, a study of the unwarranted epidemic of benzodiazepine use in Israel, found that older Arab Israelis are much less likely to receive benzodiazepine or benzodiazepine-receptor agonists than older Jewish Israelis [18]. A national Israeli study on participation in health promotion workshops has found that although participation rates among Arabs were lower than among Jews, once enrolled in a workshop, Arabs tended to attend more meetings than Jews [19].

However, it is not possible to rule out that the lack of disparities reported by Gal [2] and colleagues may be partly explained by limitations of the methodology used in that study. For example, Bago and colleagues suggest that more robust assessments that account for the individual variation over time may lead to identification of more pro rich distributions of physician utilization that can be otherwise observed [15]. Thus, utilizing additional individual covariates for which the analysis could have controlled (for example, general health status, number of additional comorbidities), could have led to the identification of gaps, which were not evident in the current analysis. Indeed, a previous study on health care utilization inequity in Israel has shown that using comprehensive health needs assessment measures allowed the study team to detect relative underutilization in use of specialist and diagnostic services, which could not be detected with less comprehensive forms of health-needs adjustments [20].

The lack of disparities reported by Feldman and colleagues [1] presents evidence for the lack of discrimination at the pediatric ED, contrary to findings on ethnic differences in ED waiting times from the US studies, which showed that Hispanic children and non-Hispanic black children had longer waiting times than non-Hispanic white children [21, 22]. The study by Feldman and colleagues is noteworthy in that it controlled for the most pertinent potential confounders for a study of waiting times - time of arrival and triage level (an indicator of the need for urgent care).

Additionally, Feldman and colleagues address a potential reason for disparities in waiting times by differentially examining waiting times by ethnic concordance. Contrary to other studies, concordance between providers and patients was not related to differentiated waiting times. For example, in a study on patient-provider language concordance and the quality of transitional care in oncology patients at the same hospital (Rambam Medical Center), Rayan et al. report that language concordance was associated with higher ratings of the care transition experience by Arab and immigrant minority groups compared with the general Hebrew speaking population [23].

## Conclusion

To conclude, the findings on the mainly lack of differences amongst Arabs and Jews in treatment of chronic comorbidities in schizophrenia patients [2] and in pediatric ED waiting times [1], reflect areas of equitable health care delivery within the Israeli healthcare system. These findings are at variance with the large body of literature that indicates the widespread disparities within the Israeli health care system.

The phenomena of health and healthcare disparities is complex and should utilize rigorous methodologies to account for the various factors that may affect the manifestation of differences amongst population groups. Rigorous methodology may help detect disparities which may otherwise be missed.
